# Dysregulation of Cell Polarity Proteins Synergize with Oncogenes or the Microenvironment to Induce Invasive Behavior in Epithelial Cells

**DOI:** 10.1371/journal.pone.0034343

**Published:** 2012-04-18

**Authors:** Samit Chatterjee, Laurie Seifried, Michael E. Feigin, Don L. Gibbons, Claudio Scuoppo, Wei Lin, Zain H. Rizvi, Evan Lind, Dilan Dissanayake, Jonathan Kurie, Pam Ohashi, Senthil K. Muthuswamy

**Affiliations:** 1 Ontario Cancer Institute, Campbell Family Institute for Breast Cancer Research, University of Toronto, Toronto, Ontario, Canada; 2 Cold Spring Harbor Laboratory, Watson School of Biological Sciences, Cold Spring Harbor, New York, United States of America; 3 Department of Thoracic/Head & Neck Medical Oncology, The University of Texas M. D. Anderson Cancer Center, Houston, Texas, United States of America; Institute of Pathology, Germany

## Abstract

Changes in expression and localization of proteins that regulate cell and tissue polarity are frequently observed in carcinoma. However, the mechanisms by which changes in cell polarity proteins regulate carcinoma progression are not well understood. Here, we report that loss of polarity protein expression in epithelial cells primes them for cooperation with oncogenes or changes in tissue microenvironment to promote invasive behavior. Activation of ErbB2 in cells lacking the polarity regulators Scribble, Dlg1 or AF-6, induced invasive properties. This cooperation required the ability of ErbB2 to regulate the Par6/aPKC polarity complex. Inhibition of the ErbB2-Par6 pathway was sufficient to block ErbB2-induced invasion suggesting that two polarity hits may be needed for ErbB2 to promote invasion. Interestingly, in the absence of ErbB2 activation, either a combined loss of two polarity proteins, or exposure of cells lacking one polarity protein to cytokines IL-6 or TNF**α** induced invasive behavior in epithelial cells. We observed the invasive behavior only when cells were plated on a stiff matrix (Matrigel/Collagen-1) and not when plated on a soft matrix (Matrigel alone). Cells lacking two polarity proteins upregulated expression of EGFR and activated Akt. Inhibition of Akt activity blocked the invasive behavior identifying a mechanism by which loss of polarity promotes invasion of epithelial cells. Thus, we demonstrate that loss of polarity proteins confers phenotypic plasticity to epithelial cells such that they display normal behavior under normal culture conditions but display aggressive behavior in response to activation of oncogenes or exposure to cytokines.

## Introduction

Asymmetric distribution of proteins within cells play critical roles during several biological processes such as secretion, absorption, directional cell migration and asymmetric cell division. This asymmetry is, in part, generated by the action of cell polarity proteins [1]. Polarity proteins are signaling scaffolding molecules that sense and provide orientation cues for cells to establish proper asymmetry and normal cell architecture [1]. In epithelial cells, the regulators of cell polarity are broadly grouped as members of the apical complex and the basolateral complex. The apical complex includes proteins such as Crumbs, PALS, PATJ, Junctional adhesion molecules (JAMs), AF-6/Afadin, and Partitioning defective (Par) complex members Par3, Par6, and atypical Protein Kinase C (aPKC). The basolateral complex includes Scribble, Discs large (Dlg) and Lethal giant larvae (Lgl) [1].

Progression of carcinoma is thought to involve both dysregulation in cellular homeostasis and changes in the extracellular microenvironment; however, the molecular mechanisms by which this cooperation takes place during carcinoma progression are poorly understood. Several recent reports have demonstrated a role for cell polarity proteins as regulators of cell and tissue architecture changes that occur during transformation and acquisition of metastatic behavior of epithelial cells in culture and *in vivo* (for reviews see [2], [3]). For example TGF**β** induced metastasis of transformed cells [4] is dependent on its ability to induce epithelial mesenchymal transition (EMT) by interacting with the Par6 polarity protein complex [5]. In *Drosophila* and mammalian epithelia, inactivation of Scribble cooperates with RasV12 to promote migration and invasion [6], [7]. Transcriptional repressors such a Zeb1, Snail and Twist regulate EMT and are implicated in metastasis [8]. Interestingly, Zeb1-induced EMT requires downregulation of a polarity protein Lgl2 [9]. Thus, there is an emerging body of evidence pointing towards an important role for cell polarity changes as regulators of invasion and metastasis.

It is possible that apical polarity proteins and basolateral polarity proteins play different roles during cancer progression. Expression of oncogenes such as ErbB2 and RasV12 in normal epithelial cells induces loss of apical polarity as monitored by disruption of tight junctions or mislocalization of apical proteins [10], [11]. However, these oncogenes do not have significant effects on basolateral polarity of epithelial cells as monitored by the changes in E-cadherin junctions [10], [11]. Consistent with lack of an effect on E-cadherin junctions, activation of ErbB2 does not induce migratory/invasive behavior in normal mammary epithelial cells [10], [12]. Furthermore, mouse models of ErbB2-induced breast cancer and Ras-induced lung or pancreatic cancer demonstrate that expression of these oncogenes in epithelial cells while inducing *in situ* carcinoma they rarely induce metastases, suggesting that additional events are likely to be required for metastatic progression [13], [14].

Both composition of the tissue matrix and presence of bone marrow derived cells have emerged to be a new class of tumor promoters. Increase in stiffness or rigidity of extracellular matrix activates epithelial cell behaviors such as invasive growth, associated with aggressive cancers [15]. Infiltration of macrophages into the primary tumor is associated with metastasis and poor patient prognosis [16]. The macrophages and tumor cells engage in a paracrine growth factor loop involving EGF and CSF1 to promote invasion in a mouse model of metastatic breast cancer. In addition to EGF, macrophages secrete multiple cytokines including TNF-α and IL-6 suggesting that macrophages in the tumor microenvironment are likely to initiate a signaling network that promotes tumor progression. Consistently, high levels serum TNF-α positively correlate with high tumor grade in breast cancer. Understanding how tumor cells acquire the ability to respond to these cytokines will have important clinical implications.

Here we demonstrate that downregulation of polarity genes in non-transformed MCF-10A cells is sufficient to initiate a synergistic interaction with cytokines, stiff ECM and ErbB2 to induce invasive behavior. Our results suggest that disruption of cell polarity proteins in tumor epithelia can prime epithelial cells for invasion by cooperating with the tumor microenvironment.

## Results

### Loss of Polarity Proteins Cooperates With ErbB2 to Promote Migration and Invasion

We have previously shown that ErbB2 requires an interaction with Par6/aPKC to disrupt apical-basal polarity and transform three-dimensional mammary acini [17]. However this was not sufficient to induce migration or invasion of MCF-10A cells [10], [17]. Here, we investigated if disruption of polarity proteins other than members of the Par6 complex cooperates with ErbB2 to induce migration or invasion.

We chose two proteins that regulate basolateral polarity, Scribble and Dlg1, and one apical protein AF-6/Afadin. Both Scribble and Dlg1 localize to E-cadherin junctions [18], whereas AF-6 localizes to apical junctions [19], [20] in polarized epithelial cells.

**Figure 1:**
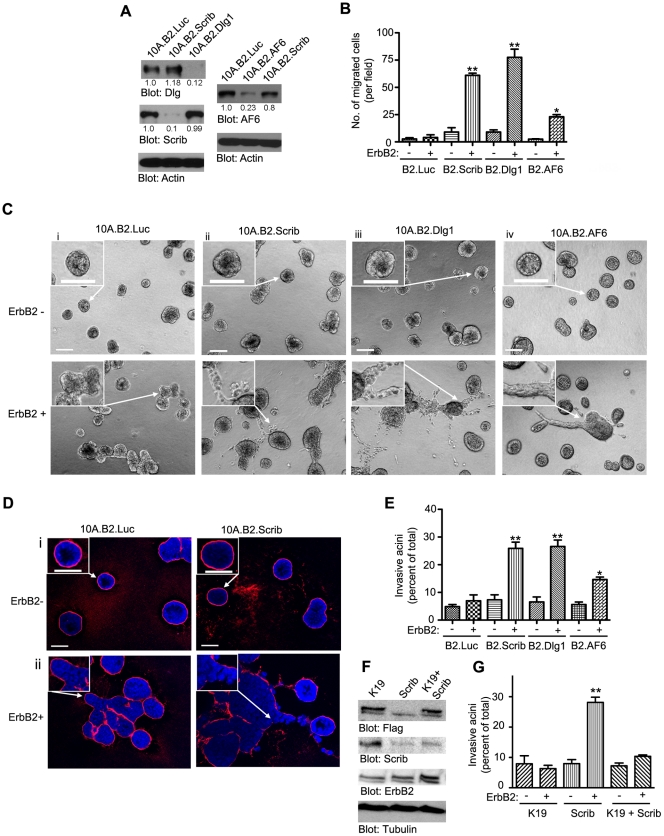
Loss of Scribble, Dlg1 or AF6 cooperates with ErbB2 activation to promote migration and invasion in MCF-10A cells. (A) Immunoblot of lysates from 10A.B2 expressing control (Luciferace, 10A.B2.Luc) or Scribble (10A.B2.Scrib) or Dlg1 (10A.B2.Dlg1) or AF6 (10B2.AF6) shRNAs. (B) Transwell cell migration assay in of cell lines in Panel A in the presence (+) or absence (−) of the ErbB2 activator. The graph represents mean of three independent experiments ± S.E.M. *, p<0.05, **, p<0.005. calculated using an unpaired t-test comparing ErbB2-activated polarity-gene knockdown cells with Luc control cells. (Ci-iv) Morphology of 3D acini derived from 10A.B2.Luc,10A.B2.Scrib, 10A.B2.Dlg1 and 10A.B2.AF6 cells grown in M/Col-I in absence (ErbB2−) or presence (ErbB2+) of ErbB2 activator. Scale bars, 100 µm. (Di-ii) M/Col-I grown 10A.B2.Luc or 10A.B2.Scrib acini treated or untreated with ErbB2 activator fixed and immunostained for Laminin (Red) and DAPI-stained for nuclei (Blue). Scale bars, 50 µm. Arrows indicate area of the image magnified in the inset. (E) Percentage of acini showing invasive protrusions were quantified and mean (± S.E.M.) plotted from at least three independent experiments. *, p<0.05 based on an unpaired t-test comparing ErbB2-activated polarity-gene knockdown B2 cells and control Luc.B2 cells. (F) Lysates from 10A.B2 and 10A.B2.Scrib transfected with Par6K19A-Flag (K19 and K19+Scrib) or untransfected (Scrib) cells were immunoblotted for Flag to show Par6.K19A overexpression. ErbB2 blot shows expression levels of ErbB2 in transfected and untransfected lines. (G) Percentage of invasive acini quantified and mean ± S.E.M. plotted for K19, Scrib and K19+Scrib cells. Note the suppression of invasion in K19+Scrib cells compared to Scrib knockdown cells. **, p<0.005 in an unpaired t-test comparing ErbB2− and ErbB2+ in Scrib knockdown cells. See Materials and Methods for details.

We used short-hairpin (sh) RNAi (see Materials and Methods for details) to stably knockdown Scribble (10A.B2.Scrib), Dlg1 (10A.B2.Dlg1) or AF-6 (10A.B2.AF-6) in MCF-10A cells expressing a chimeric ErbB2 (10A.B2) [10] that can be induced to dimerize using a small molecule ligand, AP1510 (Fig. 1A). At least two independent shRNAs were used for each gene (data shown for one shRNA and Fig. S5). In all cases we achieved more than 70% knockdown of expression (Fig. 1A, S2A). Downregulation of one polarity gene did not result in any compensatory increase in other polarity proteins under investigation or another polarity protein Par3 (Fig. 1A, S5, S6 and S7). Loss of basolateral polarity proteins Scribble or Dlg1 induced a more than 15 fold increase in cell migration in response to ErbB2 activation when compared to control shRNA (Luciferase) expressing cells (10A.B2.Luc) cells (Fig. 1B, S1A). Loss of AF-6 induced about a five-fold increase in cell migration over the migration rates observed in control cells (Fig. 1B, S1A). Thus, loss of polarity proteins cooperated with ErbB2 to induce migration and Scribble and Dlg1 cooperate with ErbB2 significantly better than AF-6.

Next we investigated if loss of polarity proteins cooperates with ErbB2 activation to induce cell invasion. ErbB2 was activated in cells grown as three-dimensional acini in either in Matrigel or in an extracellular matrix (ECM) bed made up of a 1∶1 mixture of Matrigel/collagen-I (M/Col-I). The latter condition was chosen because, recent reports have demonstrated a role for matrix stiffness in promoting invasive behavior of epithelial cells and that increasing collagen concentration to 2.0 or 4.0 mg/ml can lead to an 5–10 fold increase in rigidity compared to stiffness observed in Matrigel [15]. Activation of ErbB2 in 10A.B2.Luc cells induced formation of non-invasive multiacinar structures in both Matrigel (Fig. S1Bi) and in M/Col-I (Fig. 1Ci,E). These multiacinar structures had an intact basement membrane deposition, as monitored by Laminin V immunostaining (Fig. 1Di). However, activation of ErbB2 in 10A.B2.Scrib and 10A.B2.Dlg1 cells induced invasive growth into the surrounding matrix with basement membrane breakdown, in addition to inducing multiacinar structures (Fig. 1Cii–iii and Fig. 1Dii, Fig. 1E and Fig. S1Bii–iv) when grown on M/Col-I. Neither the invasive behavior nor the basement membrane breakdown was observed in cells grown on Matrigel (Fig. S1B) demonstrating the need for both loss of polarity proteins and a stiff ECM composed of M/Col-I for ErbB2 activation to induce invasion of MCF-10A cells. As observed in the migration assays, loss of the apical polarity protein AF-6 had weaker invasive ability compared to that observed in Scribble or Dlg1 RNAi cells (Fig. 1Civ, E) suggesting that the polarity proteins differ in the way they cooperate with ErbB2 activation.

We have previously demonstrated that ErbB2 disrupts the Par6 protein complex, and the interaction with Par6/aPKC complex is required for the ability of ErbB2 to disrupt apical-basal polarity [17]. However, as shown above, MCF-10A cells require inactivation of another polarity gene (Dlg1, Scribble or AF-6) for ErbB2-induced invasion. We tested if ErbB2 requires an interaction with Par6/aPKC complex to cooperate with downregulation of another polarity protein to induce invasion. We activated ErbB2 in 10A.B2.Scrib RNAi cells expressing Par6K19A, a Par6 mutant that does not bind aPKC and functions as a dominant interfering mutant for blocking the ability of ErbB2 to regulate the Par6/aPKC complex and apical polarity [17] (Fig. 1F). Interestingly, expression of Par6K19A significantly inhibited the ability of ErbB2 to induce invasion in cells lacking Scribble (Fig. 1G). Thus, both Par6/aPKC and loss of another polarity protein were required to induce invasive behavior.

### Loss of Cell Polarity Protein Expression and Promotion of Invasive Behavior in Multiple Cell Types

**Figure 2:**
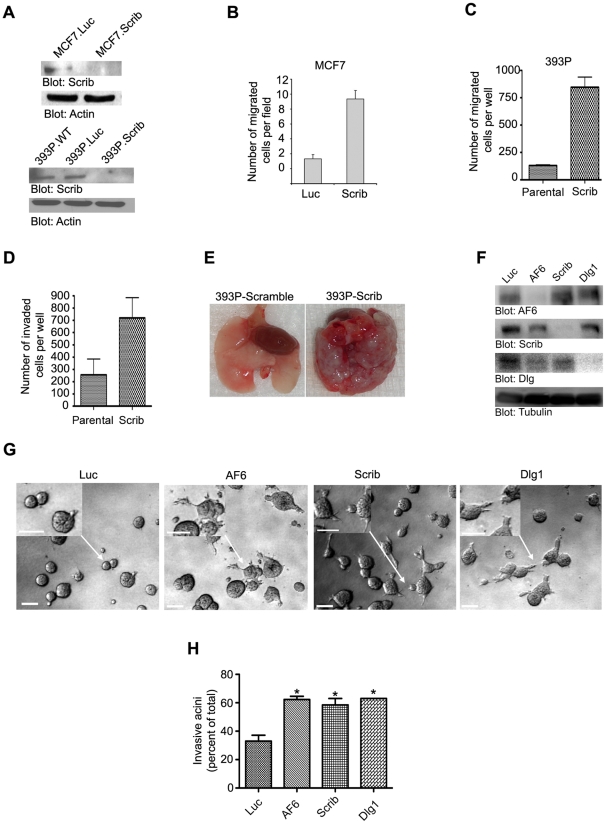
Loss of Scribble, Dlg1 or AF6 promotes invasion in multiple non-invasive transformed cell lines. (A) Control (Luc) or Scribble shRNA transfected MCF-7 (left panel) or Ras-transformed 393P cell lysates (right panel) probed for Scribble expression. (B–C) Quantification of transwell cell migration in indicated lines plotted as mean from at least three independent experiments ± S.E.M. (D) Quantification of invasion in 393P cells shows induction of invasion in Scribble knockdown (393P.Scrib) cells compared to wild-type control (393P.WT). (E) Photographs of lungs at necropsy from animals receiving tail vein injection of either 393P scramble (left) or 393P Scribble knockdown cells (right). (F) Lysates from DCIS.COM cells transfected with shRNAi for Luc, Scribble, Dlg1 or AF6 and blotted for indicated proteins. (G) Phase images of DCIS.COM cells for indicated knockdowns grown in M/Col-I showing invasive acini. Arrows indicate area of the image magnified in the inset. Scale bars, 100 µm. (H) Quantification of invasion represented as mean from three independent experiments ± S.E.M. *, p<0.05 calculated using an unpaired t-test comparing polarity-gene knockdown DCIS.COM cell lines with Luc control DCIS.COM cells. See Materials and Methods for details.

To assess whether loss of polarity protein-induced invasion is relevant in contexts other than ErbB2 activation in MCF-10A cells, we used a non-invasive tumor derived human cell line, MCF-7, and xenograft-selected MCF10AT displaying comedo-type Ductal-Carcinoma-In-Situ phenotype (DCIS.COM) cells. In addition, we also analyzed a non-invasive mouse tumor cell line 393P, derived from the lung adenocarcinomas induced by activation of K-RasG12D in p53R72H heterozygous background [21]. Downregulation of Scribble (Fig. 2A and 2E Scrib panel) in MCF7 and 393P cells induced a three to five-fold increase in their ability to migrate (Fig. 2B, C) compared to parental or Luc controls. Loss of Scribble also induced a two to three-fold increase in invasive properties in DCIS.COM (Fig. 2F and G) and 393P cells (Fig. 2D) demonstrating that loss of Scribble promoted invasive behavior to otherwise non-invasive transformed cells. These data together demonstrate that loss of Scribble induces migration and invasion in three independent tumor-derived epithelial cell lines.

To test if loss of other polarity genes also cooperated with a non-invasive transformed line, we tested AF-6 and Dlg1 knockdowns in DCIS.COM cells and compared them with Luc control cells (Fig. 2F). Both controls and polarity knockdowns show a non-invasive phenotype when grown in Matrigel alone (data not shown). However, cells grown in M/Col-I showed a two-fold increase in invasion in the polarity knockdown cells (Fig. 2G, H).

To determine if changes in polarity protein expression affects the ability of cells to display behaviors associated with metastasis in *in vivo* models, parental 393P and 393P.Scrib.RNAi cells were injected into the tail vein of non-transgenic mice. The parental 393P cells did not result in colonization and growth of cells in the lung. However, 393P.Scrib.RNAi cells were very effective in colonizing the lung demonstrating that loss of Scribble significantly increases the ability of Ras transformed cells to undergo metastasis to the lung (Fig. 2E).

Thus, permissive ECM and loss of polarity induces migration and invasive behavior to multiple transformed, non-invasive, cells.

### Synergistic Interaction Among Polarity Proteins Induces Invasive Behavior

**Figure 3:**
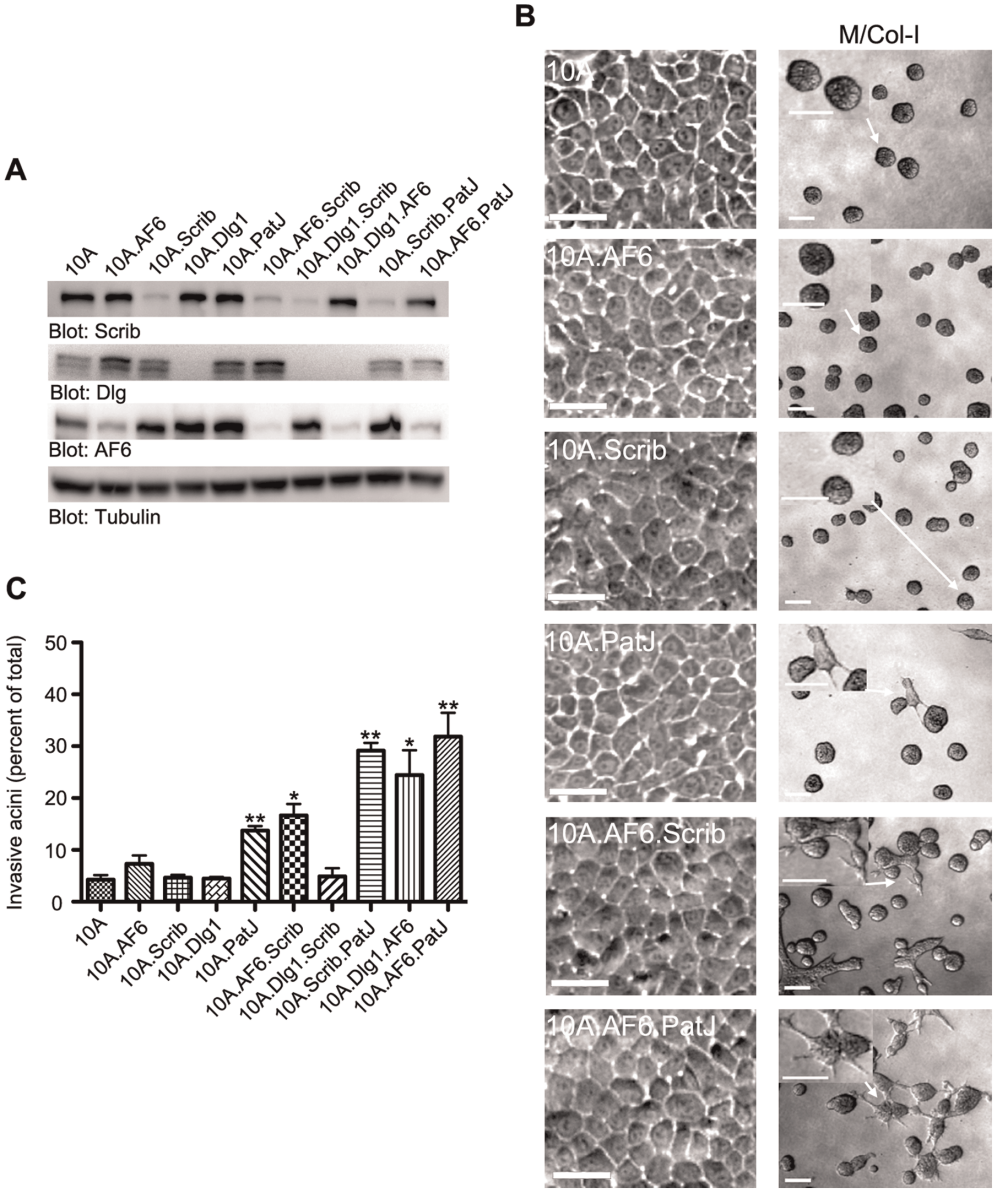
Combined loss of two regulators from apical and basal polarity complexes is sufficient to induce invasive behavior. (A) Lysates were immunoblotted to test knockdown for indicated proteins (B) Phase morphology of cells grown on plastic dishes (left panels) or M/Col-1 matrix (right panels). Also refer to *SI* Fig. S2B. Arrows indicate area of the image magnified in the inset. Scale bars, 100 µm. (C) Quantification of cell invasion plotted as mean ± S.E.M. from at least three independent experiments. *, p<0.005, **, p<0.0001 obtained in an unpaired t-test comparing indicated cell lines with parental MCF10A cells (10A). See Materials and Methods for details.

Prompted by the observation reported in Figure 1, where we found that ErbB2 requires inactivation of both Par6/aPKC and another polarity protein to promote invasive behavior, we tested the hypothesis that a combined loss of two polarity proteins can induce invasive behavior in the absence of ErbB2 activation. To test this possibility we generated parental MCF-10A cells that express shRNA targeting Scribble, Dlg1, PATJ, or AF6 either alone or in combinations (Fig. 3A and Fig. S2A). While the immunoblots in Figure 3A showed some variation in the expression level for Dlg1, this was not reproducible where its expression level in Scrib.RNAi cells were comparable 10A.Luc cells in two independent experiments (Fig. S6). In addition, we did not observe any change in expression of polarity proteins AF-6 or Par3 (Fig. S7), between cells lacking one or two polarity proteins and the parental MCF10A cells. Thus, loss one polarity protein does not lead to changes in expression of another polarity protein, among those analyzed.

Loss of one polarity gene did not induce a change in cell morphology (Fig. 3B and Fig. S2B left panels) or result in an increased invasive behavior of MCF-10A cells in 3D M/Col-I matrix (Fig. 3B and Fig. S2B right panels and Fig. 3C). Interestingly, combined loss of two polarity proteins, AF6/Scrib; Scrib/PATJ; AF6/Dlg1; and AF6/PATJ was sufficient to induce invasion as revealed by phase images (Fig. 3B, Fig. S2B indicated panels) or by laminin V breakdown (Fig. S2D) when cells were plated on a bed of M/Col-I. However, loss of two polarity proteins did not induce invasion or affect the ability of these cells to undergo normal 3D morphogenesis in Matrigel (Fig. S2C), or affect cell morphology in monolayer cultures (Fig. 3B and Fig. S2B left panels). We also did not observe an increase in expression of mesenchymal markers such as vimentin and fibronectin or a decrease in the epithelial marker E-Cadherin (data not shown) suggesting that the loss of two polarity proteins neither induced a loss in epithelial morphology nor induced changes in expression of epithelial or mesenchymal genes. We conclude that loss of two polarity proteins induced cells to acquire plasticity, which allows them to behave like mesenchymal cells in 3D M/Col-I matrix but behave like epithelia in Matrigel and monolayer cultures.

### Synergistic Interaction Between Loss of Polarity and Pro-inflammatory Cytokines

**Figure 4:**
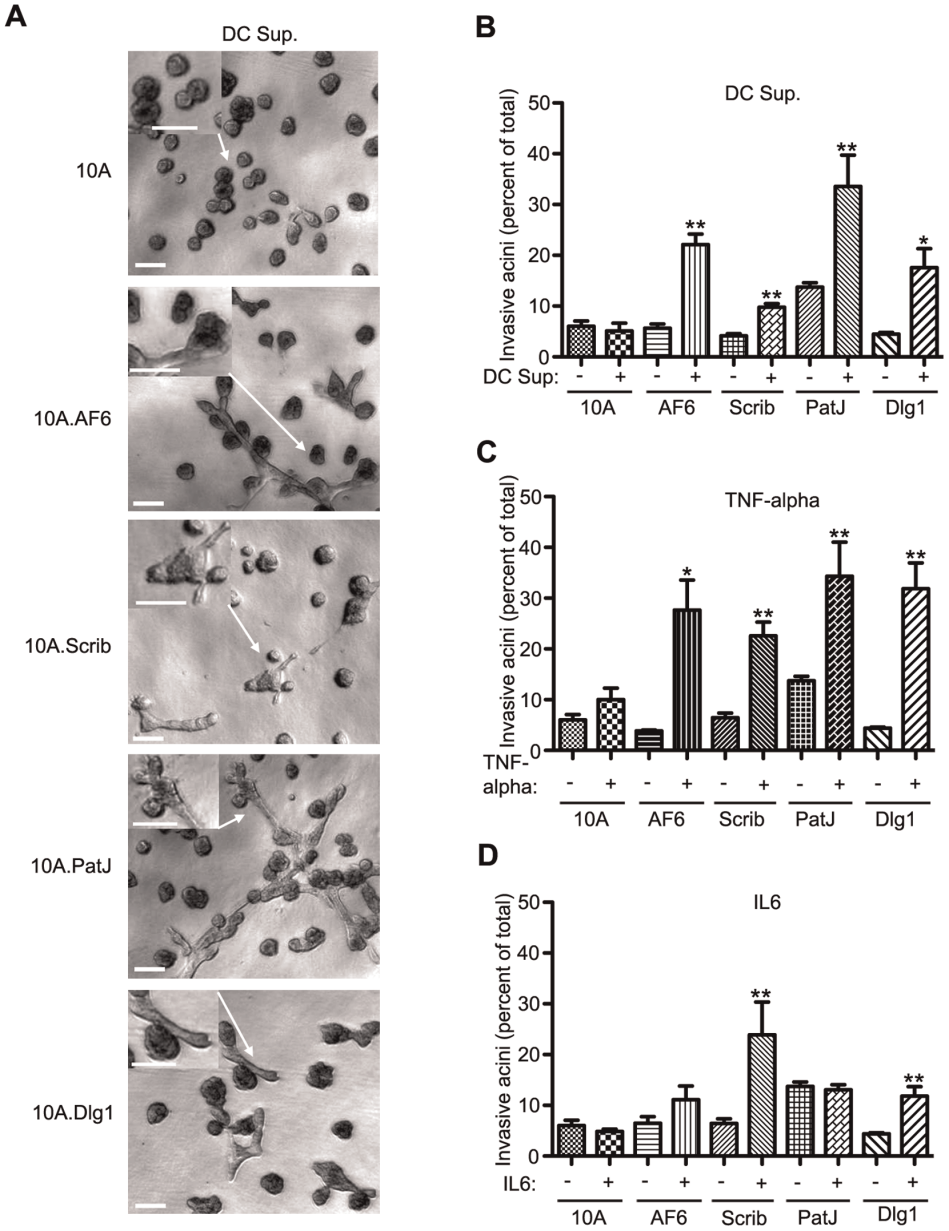
Loss of one polarity gene and cooperation with pro-tumorigenic cytokines. (A) Phase images showing induction of invasion in 4-day old acini of parental MCF10A cells or cells expressing shRNAi for indicated polarity genes growing in M/Col-I matrix and treated with 1∶6 diluted supernatant from CpG-treated dendritic cells (DC Sup.), or pro-inflammatory cytokines TNF-α (2 ng/ml) and IL-6 (25 ng/ml) (refer to *SI* Fig. S3A). Arrows indicate region of the image magnified in the inset. Scale bars, 100 µm (B) Quantification of invasion of DC Sup-treated acini plotted as mean ± S.E.M. from at least three independent experiments. (C–D) Quantification of invasion in MCF10A acini after treatment with recombinant TNF-α (C) or IL-6 (D). Also refer to SI Fig. S3B,C. *, p<0.05, **, p<0.005. p values are based on an unpaired t-test comparing cytokine-treated to the untreated values for the same knockdown.

Next we tested the possibility that pro-inflammatory cytokines could induce changes in behavior of cells lacking one polarity protein grown in Matrigel/Collagen mix. Innate immune cells such as macrophages and dendritic cells cohabit the tumor stroma and are thought to play important roles in tumor progression via secretion of pro-inflammatory cytokines such as IL-1, IL-6, TNF-α and others [22]–[24]. When macrophages or dendritic cells (DC) are treated with CpG-motif-containing bacterial oligonucleotides, they are induced to secrete IL-6 and TNF-α into the medium [25]. We reasoned that conditioned media obtained from stimulated macrophages or dendritic cells would allow us to test the interaction between macrophages and epithelial cells. Interestingly, addition of DC conditioned media diluted 1-in-6 into the MCF-10A culture media induced a significant increase in invasive structures in MCF-10A cells downregulated for expression of AF6, Scrib, PATJ or Dlg1 (Fig. 4A,B). Under these conditions we failed to observe any detectable invasive behavior in the parental MCF-10A cells demonstrating that loss of polarity protein expression was required for invasive behavior induced by activated-macrophage conditioned media.

Among the factors secreted by macrophages, IL-6 and TNF-α have important implications for breast cancer. Elevated serum levels of IL-6 correlate with the stage of metastatic breast tumor and poor patient prognosis [26], [27]. Likewise, tumor stromal TNF-α has been shown to be associated with highly invasive breast carcinoma [28], [29]. The macrophage-conditioned media used above had 12 ng/ml and 138 ng/ml of TNF-α and IL-6, respectively, as determined by an enzyme linked immunosorbent assay (ELISA) (Fig. S3A). We used recombinant IL-6 and TNF-α and determined if the concentration of IL-6 (23 ng/ml) and TNF-α (2 ng/ml) in the diluted media was sufficient to induce invasive behavior in MCF-10A cells lacking polarity proteins. Interestingly, TNF-α was effective in inducing invasive behavior of cells lacking polarity proteins but not the parental MCF-10A cells (Fig. 4C and Fig. S3B). IL-6 stimulation was less effective and only MCF-10A cells lacking Scribble and Dlg1 showed a significant response (Fig. 4D and Fig. 3SC). These results demonstrate that epithelial cells lacking polarity proteins gain the ability to invade if exposed to altered matrix and macrophage secreted cytokines.

### Downregulation of Two Polarity Proteins Induces Expression of EGFR and Activation of Akt

**Figure 5:**
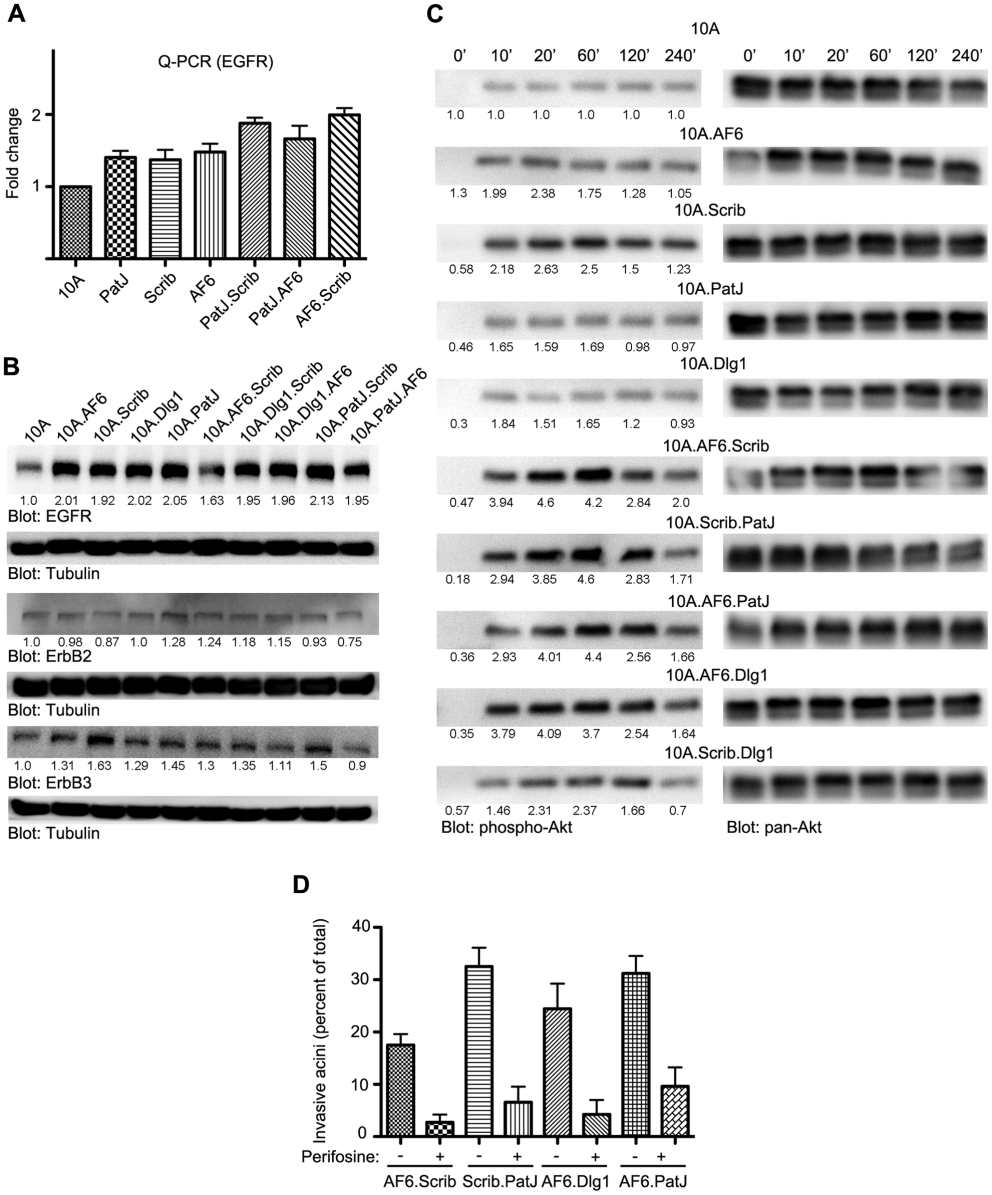
Loss of two polarity genes upregulates EGFR and activate Akt. (A–B) Parental or knockdown cell lysates were analyzed for EGFR mRNA expression (A) and immunoblotted for EGFR or ErbB2 or ErbB3. Note the increase in EGFR expression without any change in expression of ErbB2 or ErbB3 in polarity knockdown cells. (B) Parental or polarity knockdown cells were grown overnight in low-serum medium without growth factor supplements and next day replenished with Assay medium with 5 ng/ml EGF for indicated times (see Materials and Methods for details) and immunoblotted for phospho-Akt (Ser-473) first and then stripped and reblotted for pan-Akt. (D) One-day old parental or knockdown MCF10A acini were left untreated or treated with 1nM perifosine and phase images obtained and quantified after 72–96 hr post-perifosine treatment. Data represents mean ± S.E.M. from at least three independent experiments. p<0.05 obtained in an unpaired t-test comparing perifosine-treated to the untreated values for the same knockdown.

To begin to understand the mechanism by which loss of multiple polarity proteins promoted invasive behavior we investigated changes in expression of EGFR, ErbB2 and ErbB3. These receptors were chosen because MCF-10A cells are highly responsive to and require EGF ligand for growth and morphogenesis [30]. Downregulation of one or two cell polarity proteins led to a two fold increase in expression of EGFR but not ErbB2 or ErbB3 (Fig. 5A,B) demonstrating downregulation of cell polarity proteins can lead to an increase in EGFR levels. We next monitored activation of Erk1/2 and Akt kinases, two prominent signaling pathways (Ras and PI3K) downstream of EGF signaling that are known to play critical roles during cell migration/invasion. Interestingly, cells lacking two polarity proteins, with the exception of 10A.Scrib.Dlg1, showed four fold higher levels of phospho-S473 Akt compared to the parental cells in response to EGF stimulation (Fig. 5C) however, they did not differ significantly in their ability to activate Erk1/2 (Fig. S4). Among the cells lacking two polarity proteins, the 10A.Scrib.Dlg1 cells did not show invasive behavior in M/Col-I (Fig. 3C) or increased activation of Akt (S473) suggesting a relationship between increased activation of Akt and the gain of invasive behavior.

To determine if activation of Akt is required for the invasion of MCF-10A cells lacking two polarity proteins, we performed the analysis in the presence of an Akt inhibitor, perifosine. Perifosine is a synthetic alkylphospholipid that inhibits Akt [31]. Perifosine, at micromolar concentrations and via Akt-p473 inhibition, has growth inhibitory effects in many cancer cell lines including those from the breast [32]. We found that low doses (1nM) of Perifosine is sufficient to inhibit the increase in Akt473 phosphorylation (Figure S8) and treatment of cells with 1 nM Perifosine significantly inhibited invasive behavior of 3D acini derived from cells lacking two polarity proteins demonstrating that the increase in Akt activation plays an important role in promoting invasive behavior of epithelial cells lacking multiple cell polarity proteins (Fig. 5C).

## Discussion

Invasion and metastasis are the major cause of cancer-related deaths. One of the emerging themes in cancer progression is the cooperative role between cellular transformation and the tumor microenvironment that plays a critical role in promoting invasive and metastatic behavior. Changes in the tumor microenvironment, including reprogramming of stromal cells to secrete cytokines, aid in invasion and dissemination of the tumor cells. The molecular network of such cooperation is poorly understood.

In this report we demonstrate that normal epithelial cells with disruption of polarity genes cooperate with changes in the microenvironment (Matrigel/Collagen-I mixture and stimulation with inflammatory cytokines, IL-6 or TNF**α**) to gain invasive properties even in the absence of expression of transforming oncogenes. In oncogene expressing cells, loss of expression of cell polarity genes cooperates with transformation to promote invasion in culture and metastasis *in vivo*.

Our results provide a surprising new insight into the nature of collaboration between ErbB2 and disruption of polarity. Our results on cooperation between disruption of cell polarity and activation of ErbB2 are consistent with recent studies in Zebrafish where loss of Lgl2 cooperated with ErbB2 to induce invasive behavior [33] and in human breast cancer cells where overexpression of 14-3-3**ζ**,a component of polarity complexes, cooperated with ErbB2 to induce EMT and invasion [12]. We show that inhibition of the ErbB2-Par6 pathway was sufficient to block ErbB2-induced invasion, even in the presence of the second polarity gene loss, suggesting that developing ways to inhibit the ErbB2-Par6/aPKC pathway will open new avenues for blocking metastasis in ErbB2 positive cancers.

The observations on the cooperation between ErbB2 and loss of polarity can have significant clinical implications because amplification and overexpression of ErbB2 correlates with poor patient outcome in breast and multiple epithelial malignancies [34]. In addition several lines of evidence suggest that amplification of ErbB2 is an early event in tumorigenesis and not directly associated with invasive disease [35]. For example, although only 20–25% of human breast cancers fall under the ErbB2 positive subtype, more than 45% of human non-invasive breast carcinomas can possess amplified and overexpressed ErbB2 suggesting that ErbB2 overexpression is more frequently associated with non-invasive disease [35]. Results presented in this manuscript, taken together with previously published results, demonstrate that loss of polarity proteins efficiently cooperate with ErbB2 to induce invasion and suggest that dysregulation of polarity proteins can regulate metastatic progression of ErbB2 positive cancers. In addition to cooperating with ErbB2, we show that changes in polarity proteins themselves regulate the signaling by the ErbB family of receptors. Downregulation of cell polarity proteins resulted in an increased expression of ErbB1 (Epidermal growth factor receptor, EGFR) which is likely to play a role in Akt activation. We show that inhibition of Akt blocks the invasive behavior of epithelial cells lacking cell polarity proteins. Thus, a better understanding of the changes in cell polarity proteins in ErbB2 positive cancers can identify novel ways to treat and/or predict cancer progression.

Our results provide a novel perspective on the role played by cell polarity proteins in metastasis. Our results suggest that normal polarity protein function is required for maintenance of a differentiated, epithelial cell phenotype. Disruption of multiple polarity proteins in oncogene naïve, normal epithelial cells induces phenotypic plasticity where the cells behave like invasive mesenchymal cells in response to a tumor-like microenvironment, while they behave like epithelial cells under normal microenvironmental conditions. This phenotypic plasticity has been referred to as ‘partial-EMT’, or ‘metastable’ or ‘hybrid’ state [8]. Partial-EMT has been observed in basal-type breast cancers where both epithelial and mesenchymal proteins can be co-localized within single cancer cells [36]. A comprehensive analysis of the various mouse models of breast cancer show that cells in spindle cell tumors express both epithelial and mesenchymal markers, suggesting the presence of phenotypic plasticity *in vivo* [37]. Human metaplastic breast tumors that are drug resistant and exhibit some of the characteristics of spindle cell tumors also have epithelial, EMT and stem cell like characteristics compatible with the presence of phenotypic plasticity [38].

The plastic differentiation state will provide a significant advantage for migrating/invading epithelial cells. Migrating cells need to reorganize their cytoskeleton, their vesicle trafficking and vesicle targeting machinery in order to transit from apical-basal polarity to front-rear polarity [39]. During this transition polarity proteins function as core signaling proteins that are retooled and rewired [39]. For example, in glandular epithelial cells undergoing apical-basal polarization, the Par6/Par3/aPKC complex is recruited to early nascent cell-cell contacts of epithelial cells and plays a critical role during establishment of tight junctions and apical-basal polarity [40]. AF-6 regulates tight junctions via interaction with ZO-1 [19] and Scribble localizes to cell-cell junctions and regulates establishment of lateral membrane polarity [18]. In migrating cells with front-rear polarity, the Par complex localizes to the leading edge and regulates localized activation of Cdc42 and orientation of the microtubule organizing center to facilitate directional migration [41]. Scribble localizes to the leading edge and regulates assembly of β-pix and PAK1 and induces localized Rac activation and directional migration [18] and AF6 relocalizes to the leading edge of migrating fibroblasts [42]. While the mechanisms by which loss of a polarity protein regulates cell behavior is not well understood, it is likely that it involves both direct and indirect mechanisms. Loss of polarity proteins may directly affect signaling pathways since many of the polarity proteins function as signaling scaffolds that assemble multiple signaling transduction proteins. In addition, loss of one polarity protein can induce changes in localization and function (not necessarily changes in expression levels) which in turn can indirectly affect cell behavior. A deeper understanding of how changes in polarity proteins induce the phenotypic plasticity is likely to provide novel insights for understanding metastasis.

## Methods

### Antibodies and Other Reagents

Goat polyclonal antibody against Scribble and rabbit polyclonal anti-ErbB2 (Neu, C18) were obtained from Santa Cruz Biotechnology (Santa Cruz, CA). Mouse monoclonal anti-Laminin and anti-ErbB3 antibodies were obtained from Millipore (Billerica, MA). Mouse monoclonal antibodies to Dlg and AF6 were obtained from BD Biosciences (San Jose, CA). Mouse monoclonal anti-Flag, anti-tubulin and anti-actin antibodies were obtained from Sigma (St Louis, MO). Anti-HA tag monoclonal antibody was obtained from Covance (Princeton, NJ). Rabbit monoclonal antibodies to EGFR, Phospho-Akt (Ser-473), Pan-Akt, Phospho-Erk1/2, and pan-Erk were obtained from Cell Signaling Technologies (Beverly, MA). Secondary HRP-conjugated antibodies against mouse and rabbit were purchased from GE Healthcare (Buckinghamshire, UK). Secondary HRP-conjugated antibody against goat was obtained from BioRad Laboratories (Hercules, CA). Pierce Fast Western kit was obtained from Thermo Scientific (Waltham, MA). CpG Oligonucleotide (ODN 1826) was purchased from TCAG facility (Toronto, ON, Canada).

### Hairpins and Constructs

Short-hairpin RNAi sequence against Scribble and cloning into MSCV-LTR-PURO-IRESGFP retroviral expression vector has been described before [17]. Dlg1-hairpin and AF6-hairpin sequences were obtained from shRNA library (http://cancan.cshl.edu/cgi-bin/Codex/Codex.cgi) in PSM2 vector backbone. A 97-nucleotide oligonucleotide was synthesized containing a 5′ *miR30* flanking sequence, a sense strand *Dlg1 or AF6* target sequence, a common *miR30* loop sequence, an antisense strand targeting *Dlg1 or AF6 or PatJ* and a common 3′ *miR30* flanking sequence (RNAi Dlg1 sequence: GCTGTTGACAGTGAGCGCGCAGATGATGAATAGTAGTATTAGTGAAGCCACAGATGTAATACTACTATTCATCATCTGCTTGCCTACTGCCTCGGA;

RNAi AF-6 sequence:TGCTGTTGACAGTGAGCGCGGTGGAACATTGAGAATTTATTAGTGAAGCCACAGATGTAATAAATTCTCAATGTTCCACCTTGCCTACTGCCTCGGA; RNAi PatJ sequence: TGCTGTTGACAGTGAGCGCCCGATCACGCATGAGCATATTTAGTGAAGCCACAGATGTAAATATGCTCATGCGTGATCGGTTGCCTACTGCCTCGGA). The sequences were amplified using PCR primers that recognize the *miR30* flanking sequence and had *Xho*1 and *EcoR*1 restriction enzyme sites. The PCR product was subcloned into the MSCV–LTR–PURO–IRESGFP [17] or MSCV-Blasticidin vectors. Preparation of virus, infection and selection were performed as previously described [17].

### Cell Culture

MCF10A cells were maintained in DMEM/F12 with supplements, referred to as Growth medium, as previously described [10], [17]. For migration and invasion assays MCF10A cells were plated in a low-EGF (5 ng/ml) DMEM/F12 medium, referred to as Assay medium, as previously described [10], [17]. DCIS.COM cells were grown in DMEM/F12 media with 5% Horse serum. MCF-7 cells were maintained or plated for migration in MEM supplemented with 10% FBS, 10 µg/ml insulin and 1% Penstrep. MCF10A expressing FKBP-ligand-inducible chimaeric-ErbB2 (10A.B2) was constructed as described [10], [17] and ErbB2 activation induced by addition of bivalent FKBP ligand AP1510 at 1 µM concentration.

The 393P lung adenocarcinoma cell line from a *p53^R172H^* ^Δ*g/+*^ *K-ras^LA1/+^* mouse (129Sv background) has been previously described [43] and was derived from a primary lung tumor at necropsy. The tissue was minced, placed in culture, and passed serially in RPMI 1640 with 10% FBS, which yielded a mass population of tumor cells. The cells were grown in a humidified atmosphere with 5% CO_2_ at 37°C in RPMI 1640 with 5% fetal bovine serum (FBS).

### Migration Assay

10A.B2 cells were plated at a density of 5×10^5^ on 8 µm-pore transwell plates in Assay medium without EGF [44]. ErbB2 was activated (ErbB2+) by addition of AP1510 or kept inactivated (ErbB2−) by addition of solvent control as described in the preceding section. After 60 hrs cells were fixed with 5% formalin (in PBS), permeabilized with 0.5% saponin (in PBS), cells on upper surface of filter scraped with Q-tips, remaining cells labeled with DAPI, filter dislodged from the plastic insert, placed on glass slide and mounted with Vectashield (Burlingame, CA). Fluorescence images of multiple fields were obtained as described in later sections. DAPI-positive fluorescent nuclei were counted on Image-J from NIH image using “Analyze particle” routine. At least three independent experiments were quantified and data represented as mean (± S.D.). *P*-values were calculated using two-tailed unpaired t-test.

393P cells were seeded at 5×10^4^ onto Transwell plates coated with 0.1% gelatin with RPMI 1640 containing 5% FBS placed in the lower well as the chemoattractant. Each condition was performed in triplicate or quadruplicate. After 16–18 h incubation, the medium was removed and the cells fixed with 90% ethanol. The migrated cells were stained with 0.1% crystal violet, washed with ddH_2_O, and five microscopic fields counted per filter.

### Invasion Assay

MCF10A cells were grown as three-dimensional acinar structures in Matrigel:Collagen-I (M/Col-I) matrix as previously described [44]. Briefly, 8-well chamber slides were coated with 70 µL of 1∶1 mix of neutralized collagen and matrigel. MCF10A in Assay medium were seeded onto coated wells at a density of 5×10^3^ cells per well. In experiments with 10A.B2 lines, ErbB2 was activated or kept inactive by adding AP1510 or solvent control respectively to 8-day old acinar structures. Acini were monitored for invasive protrusion from day 2 to day 16 after addition of AP1510 with changes to the top medium with fresh AP1510 after every 4 days. Phase images were collected on Zeiss Axiovert 200 M using AxioVision 4.4 as described earlier [17] or Leica. MCF10A lines (10A) with indicated knockdowns were plated as above, except acini were imaged after 4 days in M/Col-I culture. Number of invasion-positive acini were counted and represented as percentage of total acini. Acini with at least one or more invasive protrusions (for examples, see Fig. S1C-D) were scored as invasion-positive. One or more acini associated with a single or multiple invasive protrusions were counted as separately invasive. Approximately 200–300 acini were quantified from two replicate wells per experiment and data from at least three independent experiments were used for calculation of mean (± S.E.M.). *P*-values were calculated using two-tailed unpaired t-test.

393P cells were seeded onto GFR-Matrigel (BD Biosciences)-coated transwell filters and invaded cells fixed and stained as in Migration assay.

### Growth Factor and Serum Starvation

Parental or polarity gene knockdown MCF10A cells were grown for four days on plastic dish in Growth medium, washed twice with PBS and fresh DMEM:F12 with 2% horse serum without growth supplements was added overnight. Next day, cells were brought out from starvation by adding Assay medium with 5 ng/ml EGF for indicated times. At the end of each time interval, cells were washed with ice cold PBS and lysed with RIPA buffer containing protease inhibitors. DNA was sheared using syringe, lysate cleared of debris by spinning and protein estimation performed according to BCA protocol. The sample buffer-treated lysate was run according to routine protein gel separation procedures.

### Immunoblotting

For transfer of proteins from gel to PVDF membrane, dry I-blot transfer (Invitrogen, Carlsbad, CA) at 20 Volts for 7 min was used, the membrane treated according to manufacturer's instructions and blocked with 5% milk in TBST. Phospho-specific antibodies or total protein antibodies were incubated overnight at 4°C, washed three-times with 5% milk in TBST, incubated with HRP-conjugated secondary antibody at room temperature for 1 hour, washed thrice and developed using standard ECL kit. Stripping of membrane was performed by incubation with 0.5 N NaOH in de-ionized water for 10 min at RT on a shaker.

### Immunofluorescence Microscopy

M/Col-Igrown MCF10A acini were fixed as described earlier [44]. Briefly, acini were fixed with 4% paraformaldehyde or 5% Formalin, quenched with PBS:Glycine, permeabilized with 0.5% TritonX-100 in PBS, blocked first in IF buffer (130 mM NaCl, 7 mM Na2HPO4, 3.5 mM NaH2PO4, 7.7 mM NaN3, 0.1% BSA, 0.2% Triton X-100, 0.05% Tween 20) plus 10% goat serum (GS) for 1–2 h and subsequently with 2° blocking buffer (IF buffer containing 10% GS and 20 mg ml-1 goat anti-mouse F(ab)′_2_) for 30–45 min. The acini were labeled with primary antibody to laminin overnight at room temperature (RT) and next day with secondary Alexa-568 for 1 hour at RT. DAPI was used for for nuclear staining.

Fluorescence images were obtained with Zeiss Axiovert 200 M using AxioVison 4.4 and ApoTome imaging system or Olympus confocal system.

### Quantitative RT-PCR

Total RNA from samples grown in monolayer culture was purified using Trizol (Invitrogen, Carlsbad, CA) according to the manufacturer's instructions and subjected to Two-Step RT-PCR analyses using SYBR® Green RT-PCR Reagents Kit on a 7500 Fast Real-Time PCR System (Applied Biosystems, Life Technologies Corporation, Carlsbad, CA), using published primers. Housekeeping gene GAPDH gene was used as an internal control for normalization of the data. Data were analyzed with the SDS 2.1 software (Applied Biosystems, Life Technologies Corporation, Carlsbad, CA). One set of primer sequences (out of three tested) for PatJ was (FORWARD: CCTGTGGATCTGCAGAAGAAAGC/REVERSE: TGATGTCGTCCTTGCGGAGG) and EGFR was (FORWARD: AAGGAGCTGCCCATGAGAAA/REVERSE: TGGCTTCGTCTCGGAATTTG).

### Preparation of Dendritic Cell Supernatant and ELISA

Supernatants from overnight cultures of dendritic cells grown with or without 10 μM CpG [45] were analyzed by enzyme-linked immunosorbent assay for the following cytokines TNF-α (BD OptiEIA Cat# 558534)), IL-6 (BD OptiEIA Cat# 555240) according to manufacturer's protocol.

### Syngeneic Tumor Cell Injections

All animal experiments were reviewed and approved by the Institutional Animal Care and Use Committee at The University of Texas M. D. Anderson Cancer Center. Wildtype 129Sv mice from our colony (males and females) of at least 8 weeks of age were used for the syngeneic tumor experiments. Animals received injections of one million cells in single-cell suspension in a volume of 100 μL of PBS or complete media by lateral tail vein. Animals were monitored regularly. There were three groups (parental cells, non-targeting vector and scribble KD), 5 animals per group. 1×10^6^ cells were injected by tail vein and the animals monitored. The first animal died at 5 weeks (in the scribble group), so the entire experiment was stopped, all the animals sacrificed and necropsy performed. The WT cells showed no lesions in the lungs by gross inspection or after H&E staining of sections and the non-targeting vector showed 3 lesions <1 mm in 2 animals and otherwise no disease. The animals receiving the scribble KD cells were all sick (weight loss, hypopnea, tachypnea) and had large, bilateral, consolidated tumors in the lungs. These were not discrete, quantifiable tumor masses, but as you see in the picture, gross overwhelming disease.

## Supporting Information

Figure S1 **Transwell migration and 3D Matrigel invasion upon loss of polarity in 10A.B2 cells and laminin breakdown in 10A.B2.Dlg1 cells.** (A) Transwell migration assay showing DAPI-stained nuclei of migrated cells for indicated knockdowns under ErbB2 inactive (ErbB2−) or activated (ErbB2+) conditions. Scale bar, 50 µm. (B) Phase morphology of 10A.B2 acini with indicated polarity gene knockdowns grown in Matrigel alone with (left panels) or without (right panels) ErbB2 activator. Note the multiacinar structures but lack of invasion in ErbB2 activated cultures. Scale bars, 100 µm. Arrows indicate region of the image magnified in the inset.(C) Immunofluorescence images showing disruption of laminin in ErbB2 activated Dlg1 knockdown 10A.B2 cells grown in M/Col-I. Scale bars, 50 µm. Examples of invasion-positive (D) and non-invasive (E) acini. See Materials and Methods for details.
(TIF)

Figure S2 **PatJ knockdown measured by quantitative RT-PCR and effect of single or combined polarity gene knockdowns grown in different culture substrates.** (A) Knockdown of PatJ was confirmed using two sets of primers (sequence of one set shown in Materials and Methods) and levels of PatJ mRNA expression in parental versus indicated polarity gene knockdown 10A cells was determined using quantitative RT-PCR. (B) Phase morphology of parental or indicated polarity gene knockdown cells grown on plastic (left panels) or M/Col-I 3D matrix. Note the absence of morphogenetic defects in plastic grown cells or lack of invasive acini in M/Col-I grown Dlg1 or Scrib.Dlg1 knockdown cells. Scale bar 100 µm. (C) Phase morphology of 10A acini with indicated knockdowns grown on Matrigel alone showing absence of invasion in both single or combined polarity gene knockdown conditions. Scale bar 100 µm. (D) Immunofluorescence images of laminin (Red) and nuclei (Blue) in acini with indicated polarity gene knockdowns grown in M/Col-I showing breakdown of laminin in invading acini only. See Materials and Methods for details.
(TIF)

Figure S3 **ELISA quantification of cytokines TNF-α and IL-6 in dendritic cell supernatant and acinar invasion in polarity knockdown 10As treated with indicated cytokines.** (A) ELISA analysis of TNF-α and IL-6 in supernatant medium of CpG-stimulated dendritic cells. (B–C) Phase images of M/Col-I grown acini of parental MCF10A cells or cells expressing sh-RNAi for indicated polarity genes showing induction of invasion in the presence of recombinant human TNF-α (B) or IL-6 (C). Scale bar 100 µm. See Materials and Methods for details.
(TIF)

Figure S4 **Lack of Erk1/2 activation in combined polarity-gene knockdowns compared to parental or single polarity gene knockdowns.** Parental or polarity-gene knockdowns cells were grown overnight in low-serum medium without growth factor supplements and next day replenished with Assay medium with 5 ng/ml EGF for indicated times (see Materials and Methods for details) and immunoblotted for Phospho-Erk1/2 and then stripped and re-blotted for pan-Erk. See Materials and Methods for details.
(TIF)

Figure S5 **Loss of AF6 or DLG1 leads to increased invasion in response to pro-tumorgenic cytokines.** A) Immunoblot for AF6 and DLG using twenty-five and 50 μg total lysates from cells transfected with AF6 or DLG1 siRNA smart pool (Dharmacon) knockdowns, four days after nucleofection with indicated amounts of siRNA in MCF10A cells grown in 2D. B) Phase contrast images of MCF10A cells grown on Matrigel:collagen five days after nucleofection with 2.0 μM siRNA pools compared to control cells. Day 4 acini have been treated with 2.0 ng/ml recombinant TNFα or 25 ng/ml recombinant IL-6 for three days.
(TIF)

Figure S6 **Effect of Scribble knockdown on Dlg expression.** Immunoblots from independent experiments showing lack of any consistent effect of Scribble knockdown on Dlg expression in MCF10A.ErbB2 (10A.B2.Scrib) cells compared to control Luc cells (10A.B2.Luc).
(TIF)

Figure S7 **Effect of polarity gene knockdowns on expression of Par3.** Lysates obtained from indicated polarity-gene knockdown cells were immunoblotted with Par3 antibody. Note the absence of any effect of knockdowns on Par3 expression.
(TIF)

Figure S8 **Effect of Perifosine treatment on AKTactivation (ser-473) in MCF10A cells.** Lysates from MCF10A wild-type (10A) or AF6 and PatJ knockdown cells (10A.AF6.PJ) were obtained as described for phospho-AKT assay (refer to Figure 5C) except one set of wells were treatedv with 1.0 nM Perfosine for the last 64–68 hrs of the assay condition. The lysates were blotted for phospho-AKT (ser-473) as described in Figure 5C and Materials and Methods.
(TIF)
