# Supplementary figures and images for: Dysregulation of Cell Polarity Proteins Synergize with Oncogenes or the Microenvironment to Induce Invasive Behavior in Epithelial Cells
Source: PLoS One. 2012 Apr 18;7(4):e34343. doi: 10.1371/journal.pone.0034343 (PMC3329530; doi:10.1371/journal.pone.0034343)

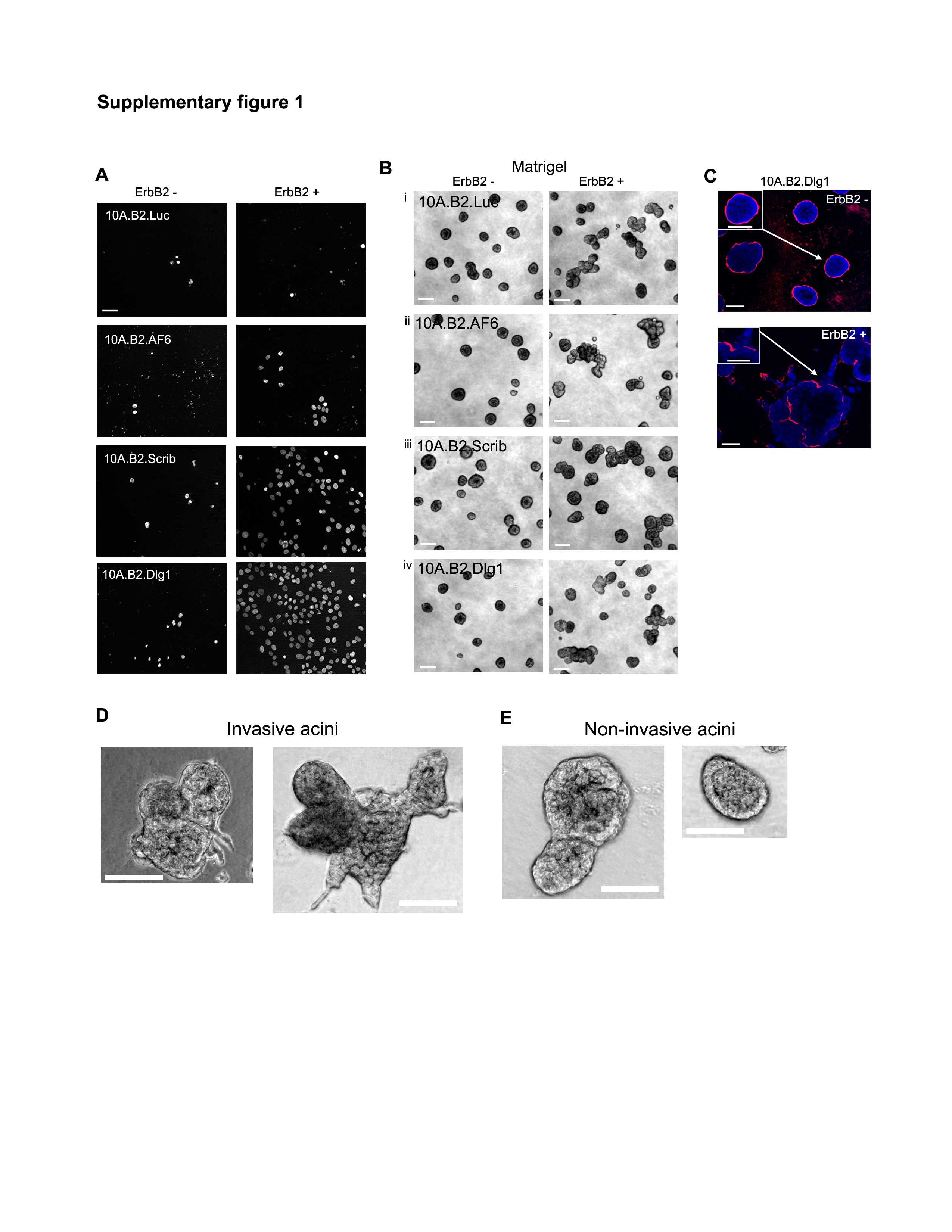

Supplement: Figure S1 — Transwell migration and 3D Matrigel invasion upon loss of polarity in 10A.B2 cells and laminin breakdown in 10A.B2.Dlg1 cells. (A) Transwell migration assay showing DAPI-stained nuclei of migrated cells for indicated knockdowns under ErbB2 inactive (ErbB2−) or activated (ErbB2+) conditions. Scale bar, 50 µm. (B) Phase morphology of 10A.B2 acini with indicated polarity gene knockdowns grown in Matrigel alone with (left panels) or without (right panels) ErbB2 activator. Note the multiacinar structures but lack of invasion in ErbB2 activated cultures. Scale bars, 100 µm. Arrows indicate region of the image magnified in the inset.(C) Immunofluorescence images showing disruption of laminin in ErbB2 activated Dlg1 knockdown 10A.B2 cells grown in M/Col-I. Scale bars, 50 µm. Examples of invasion-positive (D) and non-invasive (E) acini. See Materials and Methods for details. (TIF) [file pone.0034343.s001.tif]

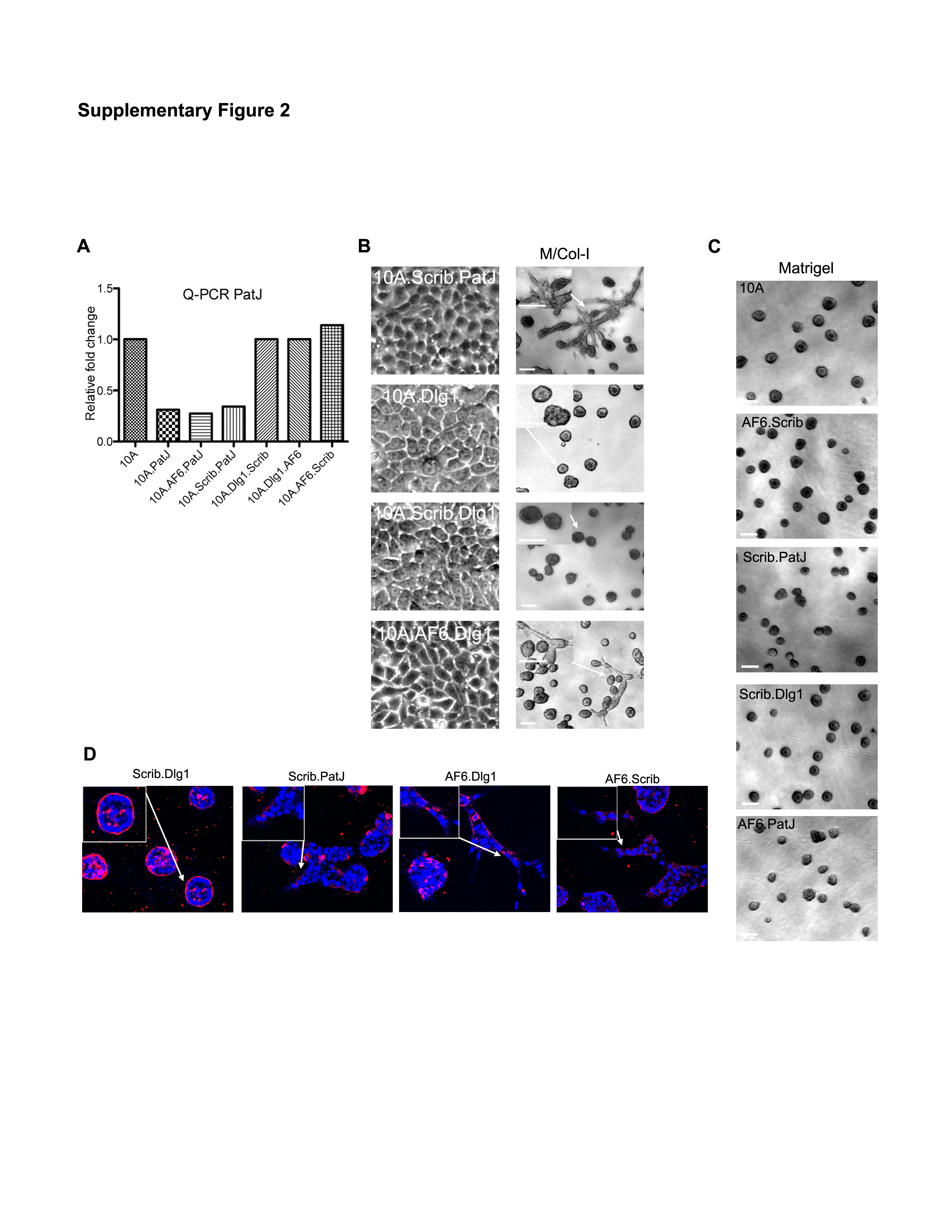

Supplement: Figure S2 — PatJ knockdown measured by quantitative RT-PCR and effect of single or combined polarity gene knockdowns grown in different culture substrates. (A) Knockdown of PatJ was confirmed using two sets of primers (sequence of one set shown in Materials and Methods) and levels of PatJ mRNA expression in parental versus indicated polarity gene knockdown 10A cells was determined using quantitative RT-PCR. (B) Phase morphology of parental or indicated polarity gene knockdown cells grown on plastic (left panels) or M/Col-I 3D matrix. Note the absence of morphogenetic defects in plastic grown cells or lack of invasive acini in M/Col-I grown Dlg1 or Scrib.Dlg1 knockdown cells. Scale bar 100 µm. (C) Phase morphology of 10A acini with indicated knockdowns grown on Matrigel alone showing absence of invasion in both single or combined polarity gene knockdown conditions. Scale bar 100 µm. (D) Immunofluorescence images of laminin (Red) and nuclei (Blue) in acini with indicated polarity gene knockdowns grown in M/Col-I showing breakdown of laminin in invading acini only. See Materials and Methods for details. (TIF) [file pone.0034343.s002.tif]

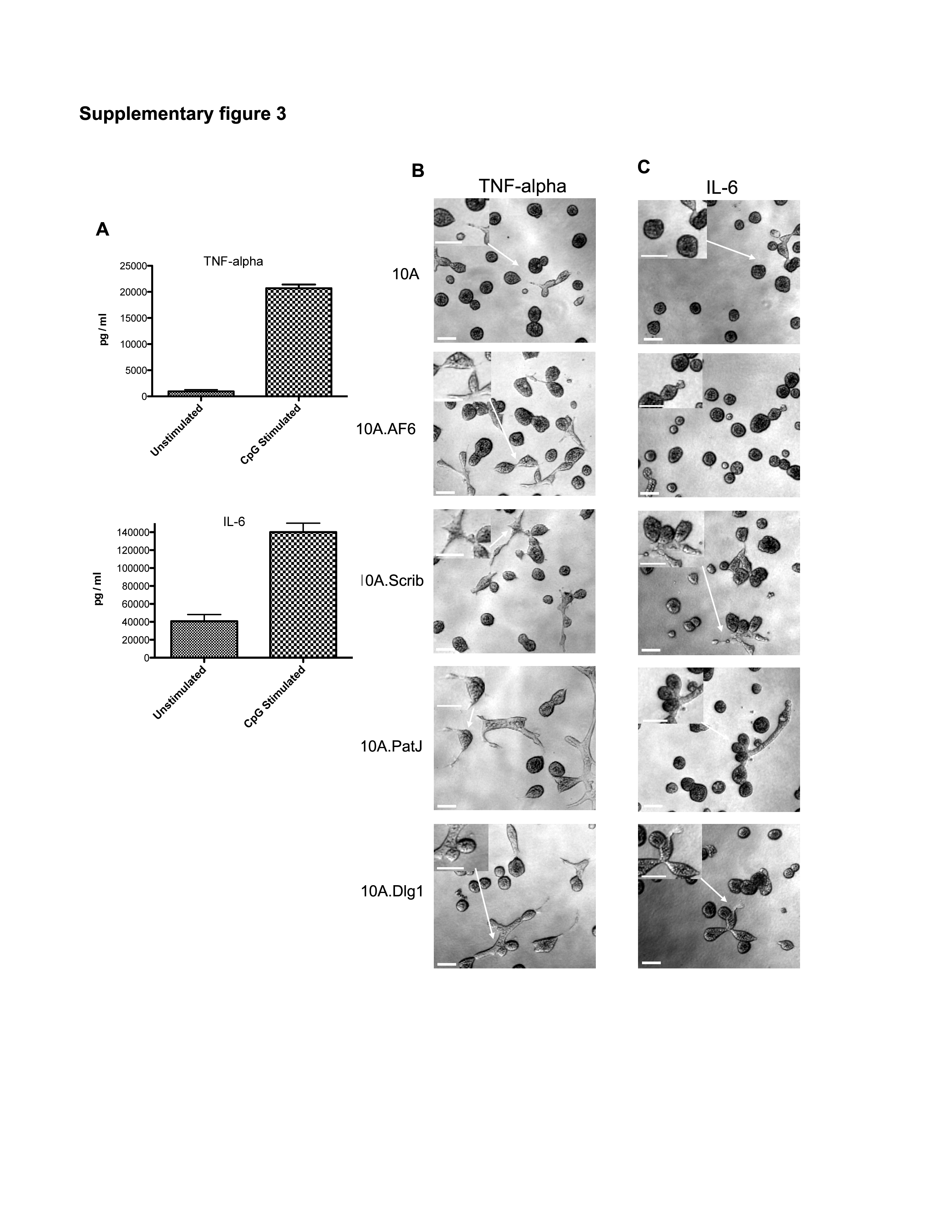

Supplement: Figure S3 — ELISA quantification of cytokines TNF-α and IL-6 in dendritic cell supernatant and acinar invasion in polarity knockdown 10As treated with indicated cytokines. (A) ELISA analysis of TNF-α and IL-6 in supernatant medium of CpG-stimulated dendritic cells. (B–C) Phase images of M/Col-I grown acini of parental MCF10A cells or cells expressing sh-RNAi for indicated polarity genes showing induction of invasion in the presence of recombinant human TNF-α (B) or IL-6 (C). Scale bar 100 µm. See Materials and Methods for details. (TIF) [file pone.0034343.s003.tif]

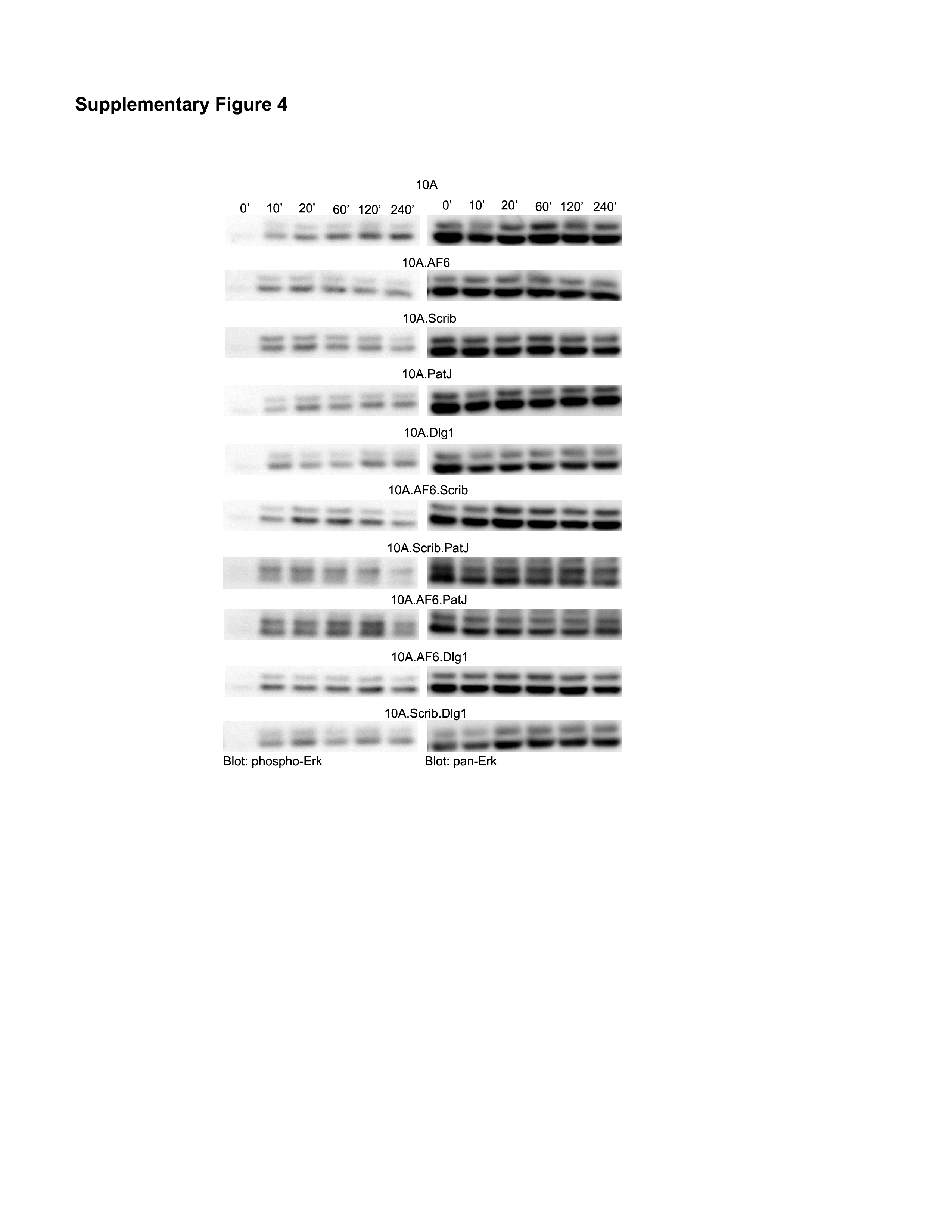

Supplement: Figure S4 — Lack of Erk1/2 activation in combined polarity-gene knockdowns compared to parental or single polarity gene knockdowns. Parental or polarity-gene knockdowns cells were grown overnight in low-serum medium without growth factor supplements and next day replenished with Assay medium with 5 ng/ml EGF for indicated times (see Materials and Methods for details) and immunoblotted for Phospho-Erk1/2 and then stripped and re-blotted for pan-Erk. See Materials and Methods for details. (TIF) [file pone.0034343.s004.tif]

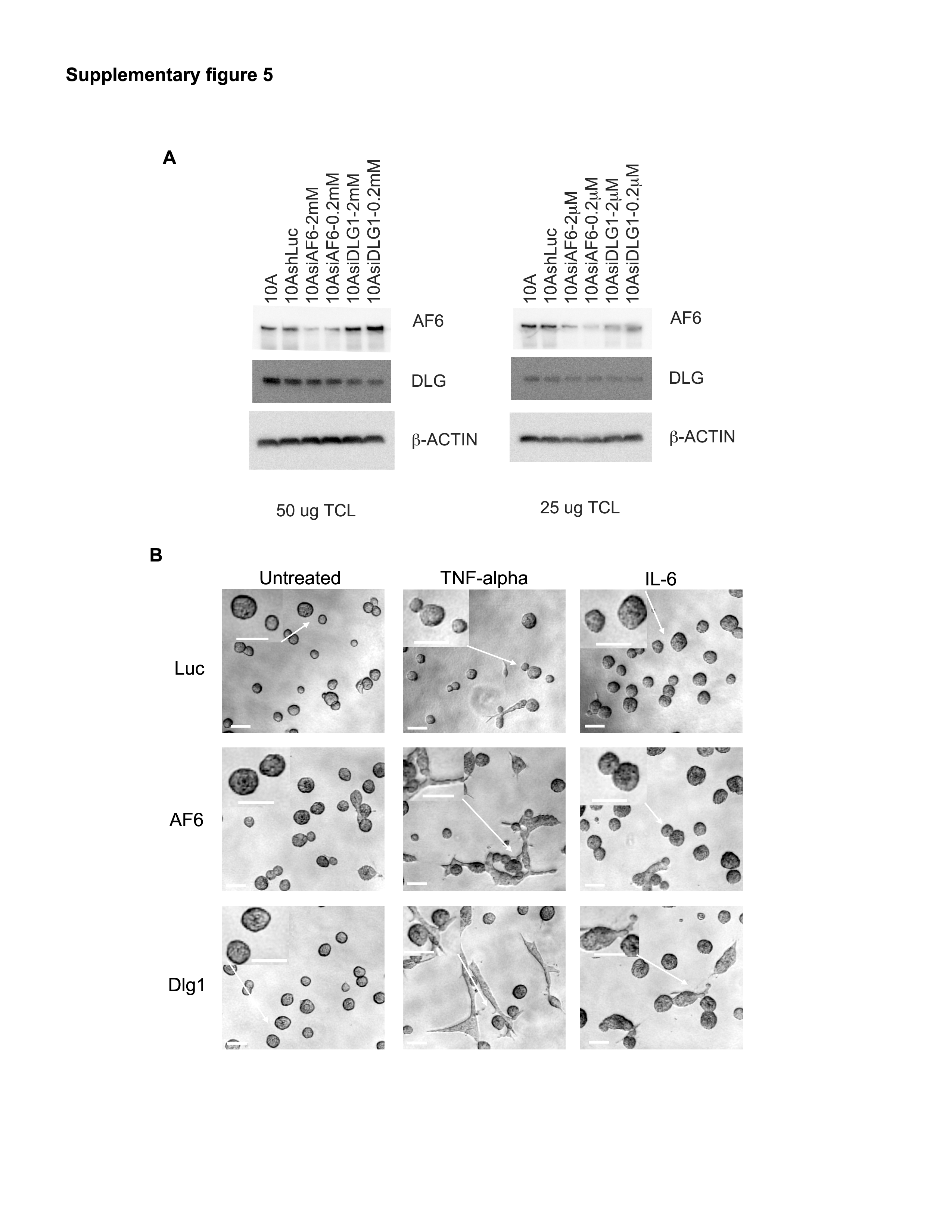

Supplement: Figure S5 — Loss of AF6 or DLG1 leads to increased invasion in response to pro-tumorgenic cytokines. A) Immunoblot for AF6 and DLG using twenty-five and 50 μg total lysates from cells transfected with AF6 or DLG1 siRNA smart pool (Dharmacon) knockdowns, four days after nucleofection with indicated amounts of siRNA in MCF10A cells grown in 2D. B) Phase contrast images of MCF10A cells grown on Matrigel:collagen five days after nucleofection with 2.0 μM siRNA pools compared to control cells. Day 4 acini have been treated with 2.0 ng/ml recombinant TNFα or 25 ng/ml recombinant IL-6 for three days. (TIF) [file pone.0034343.s005.tif]

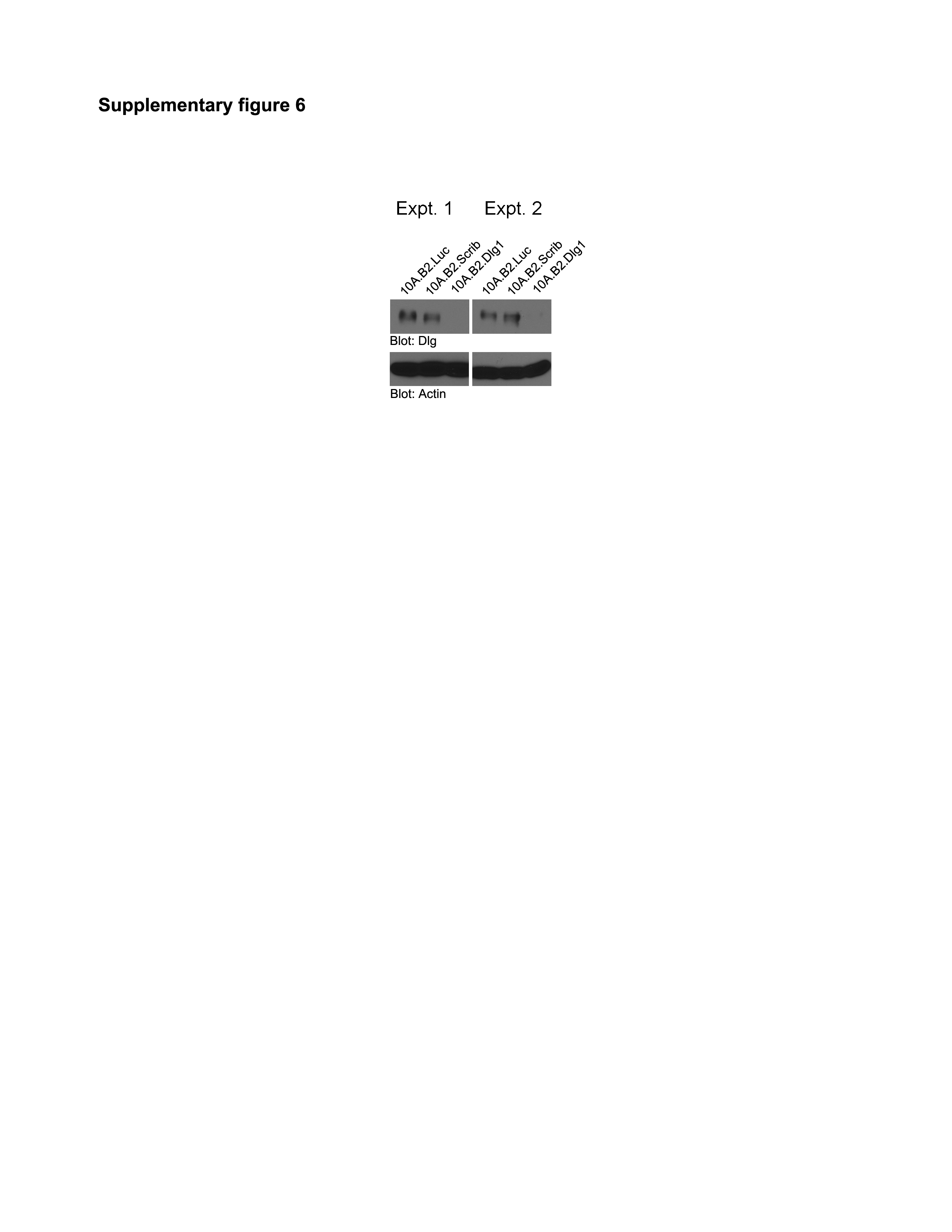

Supplement: Figure S6 — Effect of Scribble knockdown on Dlg expression. Immunoblots from independent experiments showing lack of any consistent effect of Scribble knockdown on Dlg expression in MCF10A.ErbB2 (10A.B2.Scrib) cells compared to control Luc cells (10A.B2.Luc). (TIF) [file pone.0034343.s006.tif]

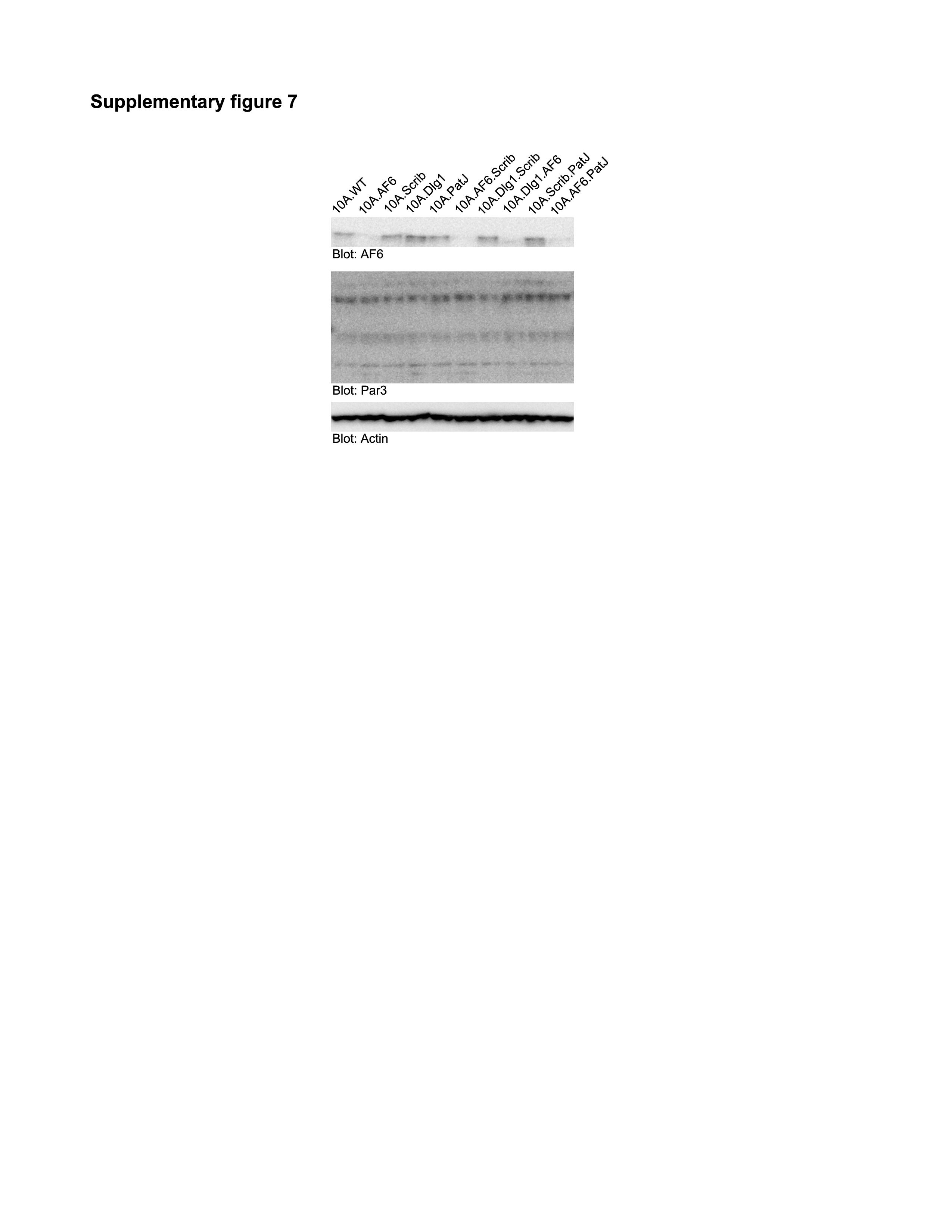

Supplement: Figure S7 — Effect of polarity gene knockdowns on expression of Par3. Lysates obtained from indicated polarity-gene knockdown cells were immunoblotted with Par3 antibody. Note the absence of any effect of knockdowns on Par3 expression. (TIF) [file pone.0034343.s007.tif]

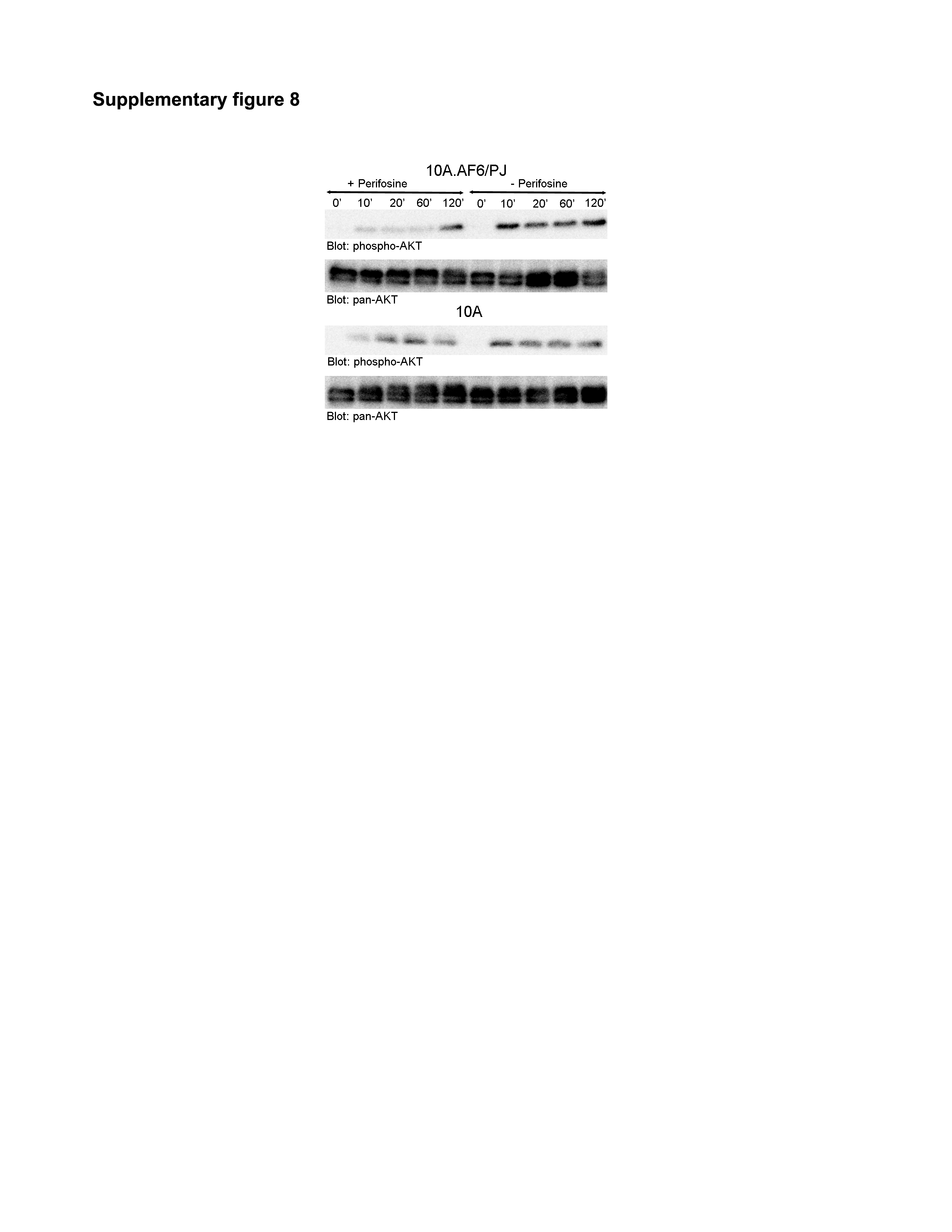

Supplement: Figure S8 — Effect of Perifosine treatment on AKTactivation (ser-473) in MCF10A cells. Lysates from MCF10A wild-type (10A) or AF6 and PatJ knockdown cells (10A.AF6.PJ) were obtained as described for phospho-AKT assay (refer to Figure 5C) except one set of wells were treatedv with 1.0 nM Perfosine for the last 64–68 hrs of the assay condition. The lysates were blotted for phospho-AKT (ser-473) as described in Figure 5C and Materials and Methods. (TIF) [file pone.0034343.s008.tif]
